# A Counterblast against Tobacco

**Published:** 1853-07

**Authors:** 


### ARTICLE XI.

### A Counterblast against Tobacco.

Amongst the numerous indications of advanced civilization that strike every observer of modern English society, the more temperate habits of all ranks is not the least remarkable. This is a gratifying feature, and has materially tended to diminish crime and misdemeanor throughout the community. But however satisfactory the present moderation in the use of intoxicating drinks, especially by the upper classes, must appear, if compared with times not very remote, all will acknowledge another vice has recently become much more common than previously, which is quite as great an abomination. We allude to the augmented consumption of tobacco, particularly the custom of smokingt hat noxious weed, now overrunning the land like a pestilence. These are strong words; but knowing the injurious effects tobacco produces upon the human constitution, both in body and mind, we feel impelled, as guardians of public health, to warn our countrymen of the dangerous consequences incurred, should they fall under the spell of this narcotic, which ought to be classed in the same category with opium, the great bane of eastern nations.

Ever since the first introduction of smoking into England, by Sir Walter Raleigh, the habit has been invariably condemned, except by its enslaved votaries. Various authors might be quoted in support of this opinion—nay, royalty itself has deigned to enter the lists of controversy, in the person of James I, when he wrote "A Counterblast against Tobacco." Amurath, one of the sultans of Constantinople, was also a decided opponent, and made it penal to smoke tobacco, on the plea that it diminished population. Again, in England, an edict was published, during the reign of Queen Elizabeth, prohibiting its use; whilst even popes anathematized those of the faithful who presumed to smoke in churches. Besides these authorities, moralists, religious teachers and aristocratic arbiters of fashion, have uniformly censured the custom as vulgar; and, like ardent spirits, all have viewed it as truly another form of dissipation. Into the evil consequences of this objectionable practice, which every day becomes more general, especially since so many foreign visitors have arrived in the metropolis, space prevents our entering at much length; nevertheless, from our experience, and having often seen cases where serious disease was occasioned, chiefly, if not entirely, by the use of tobacco, we cannot do otherwise than denounce its employment. Irrespective, however, of individual illustrations, impartial searchers after truth need only look at the condition of those countries whose inhabitants have become Nicotian worshipers. Spain, at one time a great nation, has been as if paralyzed since Toledo brought the weed from America; Holland, instead of remaining the leading commercial state of Europe, has fallen very low in the scale, being now generally enveloped in fogs and tobacco-smoke, ever redolent of Schiedam; Germany, after being the cradle of the Reformation, and notwithstanding its sending forth many pioneers of modern learning, with its "SCHNAPPS, RAUCH, and STUBENOFEN," is now chiefly known as the hot-bed of mesmerism, homeopathy, water-cures, and various other modern, yet equally absurd fancies. Italy, the birth-place of CÆSAR, DANTE, and GALLILEO—three of the greatest intellects the world ever saw, but still the land of poetry, music and song—has also fallen under the influence of the same benumbing plant; and lastly, our lively neighbors on the other side of "la manche," have, like others, become followers, in too many instances, of this filthy custom. Without going farther for proofs of these assertions, an inquirer need only walk along Piccadilly on shilling days of the Exhibition, from five to six o'clock in the afternoon, when hundreds, nay, thousands of bearded foreigners, may be seen on the tops of omnibusses, or filling the crowded streets, smoking cigars; of which they had been deprived during their stay at the Crystal Palace. The atmosphere thus becomes often so tainted, that, on regarding this strange scene, a spectator might almost believe he was in a Dutch or German Kaffe-Haus, were it not for the presence of horses, and the noise of carriages, which disperse the delusion.

How men of intellect and education, or those engaged in mental pursuits, besides persons frequenting female and well-bred society, can deliberately adopt such abominations, seems indeed surprising. Individuals who pass their time in physical labor, and only eat or sleep during the remainder, might perhaps be excused when occasionally giving way to such temptations; but for professional people, or gentlemen, to become devotees to such unseemly habits, is altogether inexcusable. Temperance leagues and kindred societies have been instituted, and often have accomplished much good, in their respective spheres; but seeing the principles of action influencing the above meritorious associations are involved in the motives which should prompt a crusade against the now more than ever prevalent use of tobacco, it is singular that parties advocating abstinence from spirituous liquids, and the disuse of sugar produced by the aid of human bondage, should not have already united their energies, in order to put down a vice which encourages the worst form of slave-labor, and also engenders a species of intoxication. This anomaly is however only one of the paradoxes met with in our free country, where some zealots will choke upon a gnat, after having swallowed an entire camel.

Although advantageous to many of our transatlantic brethren who extensively cultivate this poisonous plant; to shipowners, by whom it is brought to Europe; to our chicory chancellor of the exchequer, whereby his budget is most amply replenished; besides proving prolific in producing disease, and hence most profitable to medical practitioners; its attendant evil consequences are so numerous, that all right-thinking men must zealously oppose the spreading of a custom which fashion sanctions, but reason and experience strongly condemn. We sincerely welcome every foreign visitor now within these shores, trusting all may be pleased with their reception, and will see much which meets their approval, besides hearing the opinions entertertained by intelligent Englishment; whilst at the same time the denizens of Great Britain shall learn many things from their intercourse with strangers belonging to different climes. Expecting this result may supervene, and hoping the people of England will adopt whatever is really good in the manners peculiar to other lands, they ought never to disfigure the human face divine by those hirsute appendages, at present so often seen in London thoroughfares; and further, to quote the expressive although undignified words of King JAMES, contained in his "Counterblast," they must not convert "their mouths into smoking, sooty chimneys." To do these things would be indeed making progress backwards, instead of following the onward movement of civilization. Much more might be easily advanced, and cogent arguments stated regarding the noxious custom here denounced, but it seems superfluous. We therefore conclude our remarks by saying, in the classical language of CAMDEN—a great opponent to the use of tobacco—that the habit of smoking this weed may so seriously affect the physical and mental constitution of man, as to cause "*Anglorum corpora in barbarorum naturam degenerare.*"—*London Lancet.*

